# Distinguishing atypical parotid carcinomas and pleomorphic adenomas based on multiphasic computed tomography radiomics nomogram: a multicenter study

**DOI:** 10.3389/fonc.2025.1625487

**Published:** 2025-08-01

**Authors:** Lin-Wen Huang, Jian-Chao Liang, Pei-Kun Cai, Zhi-Ping Cai, Mei-Lin Chen, Jia-Wei Pan, Yong-Feng Wen, Yun-Jun Yang, Zhen-Yu Xu, Ya-Bin Jin, Zhi-Feng Xu

**Affiliations:** ^1^ Department of Radiology, the First People’s Hospital of Foshan, Foshan, Guangdong, China; ^2^ Department of Radiology, Zhuhai People’s Hospital (Zhuhai hospital affiliated with Jinan University), Zhuhai, Guangdong, China; ^3^ Department of Radiology, The Maoming People ‘s Hospital, Maoming, Guangdong, China; ^4^ Department of Radiology, Shunde Hospital, Southern Medical University (The First People’s Hospital of Shunde), Shunde, Foshan, Guangdong, China; ^5^ Department of Information System, the First People’s Hospital of Foshan, Foshan, Guangdong, China; ^6^ Department of Radiology, The Third Affiliated Hospital of Sun Yat-Sen University-Yuedong Hospital, Guangzhou, Guangdong, China; ^7^ Department of Radiology, Foshan Fuxing Chancheng Hospital, Foshan, Guangdong, China; ^8^ Clinical Research Institute, The First People’s Hospital of Foshan, Foshan, Guangdong, China

**Keywords:** radiomics, nomogram, multiphasic CT, parotid carcinoma, pleomorphic adenoma

## Abstract

**Objective:**

This study aimed to develop, validate, and test a comprehensive radiomics prediction model using clinical data and contrast-enhanced multiphasic computed tomography (CT) scans for differentiating between atypical parotid carcinomas (PCAs) and pleomorphic adenomas (PAs) within a multicenter cohort.

**Materials and methods:**

The study involved 218 patients diagnosed with either PAs (n=162) or atypical PCAs (n=56) (no invasion of adjacent tissues or lymph node metastases) across three anonymized hospitals, divided into a training set (n=175) and a validation set (n=43). Clinical features and radiological findings were used to develop a clinical model. Radiomics features were extracted from multi-phase contrast-enhanced CT, with feature selection achieved through statistical methods and the least absolute shrinkage and selection operator (LASSO). Radiomics signature were developed using a Light Gradient Boosting Decision Tree (LightGBM) model. A radiomics nomogram integrating significant clinical risk factors with the radiomics signature was created, with external validation conducted on an independent dataset of 32 patients from two additional hospitals.

**Results:**

In the training set, the multiphase models (model_A+P_, model_A+V_ and model_A+P+V_) demonstrated significantly superior predictive performance compared to the arterial-phase-only model (model_A_) (DeLong’s test, p=0.04–0.02). However, no significant differences emerged between the models in the validation or independent testing sets (p > 0.05). Based on recall and F1-score evaluations in the independent testing set, model_A+P_ was selected for integration with clinical risk factors to develop a radiomics nomogram. This nomogram demonstrated excellent diagnostic performance, achieving AUCs of 1.000 (training), 0.854 (validation) and 0.783 (independent testing), accuracies of 1.000, 0.864 and 0.750, and F1-scores of 1.000, 0.914 and 0.826, respectively. Key discriminative features — cluster shade, run-length non-uniformity and first-order mean, extracted via wavelet or exponential filters — significantly differentiated atypical PCAs from PAs.

**Conclusion:**

The CT-based radiomics nomogram, supplemented by machine learning, effectively differentiates atypical PCAs from PAs, presenting a non-invasive diagnostic tool that could guide treatment decisions and reduce the need for invasive procedures.

## Introduction

Salivary gland tumors, although relatively uncommon, predominantly arise in the parotid glands. Over 80% of these tumors are benign (1). The treatment strategies for benign and malignant parotid tumors (MPTs) differ significantly. Benign parotid tumors (BPTs) are generally managed with local or superficial parotidectomy, whereas MPTs necessitate a more aggressive treatment, including total or subtotal parotidectomy, potentially accompanied by facial nerve resection or postoperative chemoradiation. Thus, precise preoperative diagnosis is pivotal for selecting the appropriate treatment modality and for prognostic assessment. Fine needle aspiration biopsy (FNAB) is widely recognized as the gold standard for diagnosing parotid gland tumors. Despite its high overall accuracy, sensitivity, and specificity nearly 90% (2, 3), FNAB is invasive and associated with complications. Notably, between 5.4% and 19.4% of patients may experience facial palsy as a complication (4). FNAB also faces challenges in sampling tumors located in the deep lobe due to the procedure’s invasiveness. The inherent heterogeneity of tumors may not fully capture. When considering ‘inconclusive’ reports, the sensitivity of FNAB for detecting malignant lesions drops significantly to 48% (5). Additionally, distinguishing between malignant parotid tumors (MPTs) and benign parotid tumors (BPTs) presents significant heterogeneity, as evidenced by a large meta-analysis (6).

The most prevalent BPT is pleomorphic adenoma (PA), followed by Warthin’s tumor (WT) (1, 7). WT is characterized by significant clinical and imaging features. It predominantly occurs in elderly men with a smoking history (7), manifests as multiple nodules in both parotid glands, typically situated in the posterior lower pole of the superficial lobe (8, 9), and exhibits a rapid enhancement and washout pattern on multi-phase enhanced CT scans (10). Consequently, precise preoperative differentiation between PAs and parotid carcinomas (PCAs) becomes critically important in clinical practice. Distinguishing between PCAs and PAs based solely on clinical symptoms, such as pain and palpable masses, proves challenging. Meta-analyses indicate that while conventional imaging techniques offer high specificity, their sensitivity in differentiating between BPTs and malignant parotid tumors (MPTs) is comparatively low (11).

Magnetic resonance imaging (MRI) is favored for its superior tissue contrast and multi-parametric capabilities, establishing it as the preferred method for diagnosing PTs. Recent advancements in MRI techniques, including diffusion-weighted imaging (DWI), arterial spin labeling (ASL), diffusion kurtosis imaging (DKI), and dynamic contrast-enhanced (DCE) MRI, have been explored for PT diagnosis (12, 13). However, MRI presents several key limitations: (1) Longer acquisition times increase patient discomfort and motion artifact risk; (2) Higher costs and lower availability limit accessibility; (3) Contraindications exist for patients with claustrophobia, non-cooperation, or incompatible implants; and (4)Debate persists regarding the repeatability and diagnostic accuracy of some advanced techniques, particularly in PCAs with highly cellularity (13). Computed tomography (CT), in contrast, offers distinct practical advantages for preoperative evaluation: (1) Faster scan times improve patient tolerance, especially for uncooperative or dyspneic individuals; (2) Lower cost and wider availability enhance accessibility; and (3) It avoids MRI-specific contraindications like claustrophobia. Multi-phase enhanced CT effectively captures tumor vascular perfusion information. Critically, CT radiomics presents a unique opportunity: it can be retrospectively applied to existing multi-phase CT datasets. This is particularly valuable for analyzing parotid masses discovered incidentally. Nevertheless, CT has significant drawbacks: (1) Ionizing radiation exposure, especially concerning with multi-phase protocols as used in this study; and (2) Overlapping imaging features between PCAs and PAs (e.g., size, shape, cystic change, calcification, enhancement) frequently complicate diagnosis (10), particularly for atypical PCAs without invasion or metastasis.

Radiomics, distinguished by its ability to extract high-throughput quantitative data from medical images non-invasively, has emerged as a groundbreaking field in precision medicine. This approach, which aims to elucidate tumor heterogeneity, has gained widespread recognition for its applications in tumor classification (14, 15), outcome prediction (16), and therapy response assessment (16). Notably, radiomic models have demonstrated significant diagnostic value in differentiating between benign and malignant lympho-associated PTs (17), as well as in distinguishing between PAs, WTs (18), and basal cell adenomas (19) based on CT images. However, the rarity of PTs and the absence of a large database have limited most radiomic studies on these tumors to single-center efforts with small sample sizes. Variability in the selection of enhancement phases has led to significant discrepancies and controversies in findings. Furthermore, the stability and robustness of existing models remain suboptimal (20), posing challenges for clinical application. To date, studies have generally grouped WTs and PAs into BPTs for differentiation from malignant counterparts, with no multicenter studies specifically addressing PAs and PCAs (especially atypical PCAs) differentiation reported. Atypical parotid gland cancer refers to tumors that do not show significant malignant features, such as invasion of adjacent tissues or lymph node enlargement, on radiological imaging. Such cancers are often misdiagnosed as PAs in clinical practice. Consequently, the effectiveness of phase selection, feature extraction methods, model construction techniques, or their combinations in enhancing differential diagnosis performance remains unexplored.

This study aims to develop and validate a machine learning prediction model that leverages multi-phase CT radiomics features, clinical-radiological characteristics, and their integration to ascertain its efficacy in distinguishing atypical PCAs from PAs.

## Method

### Patient cohort

**Figure 1:**
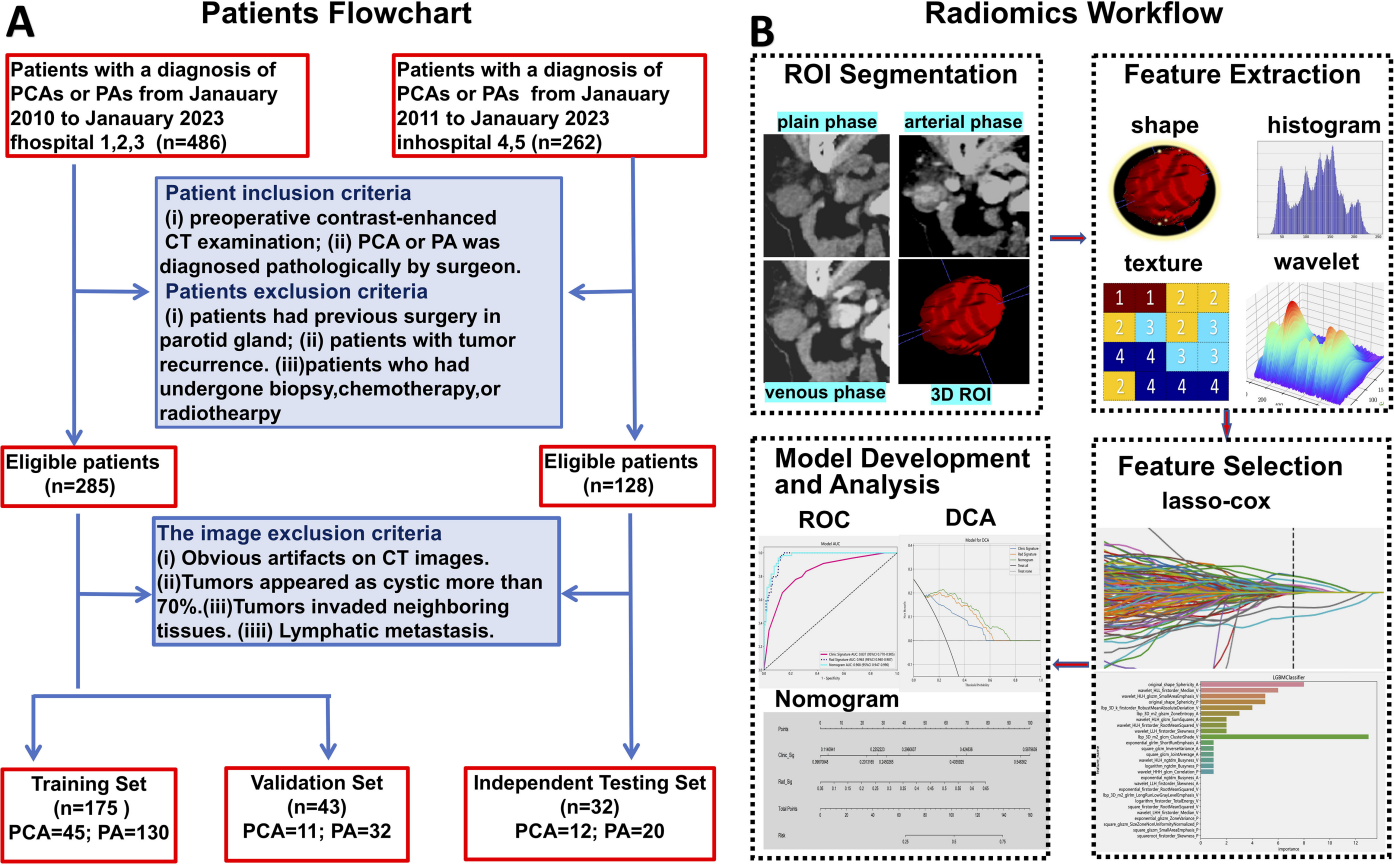
Workflow of patients recruitment **(A)**, and the radiomics analysis **(B)**.

Our study enrolled patients diagnosed with PAs or PCAs across multiple centers from January 2011 to January 2023. A retrospective collection yielded 486 patients from Hospitals 1, 2, and 3, and 262 patients from Hospitals 4 and 5. The inclusion criteria were as follows: (1) PAs or PCAs diagnosis confirmed by postoperative pathology; (2) Completion of multi-phase contrast-enhanced CT scans within one week prior to surgery. Exclusion criteria for patients included: (1) Previous parotid gland surgery; (2) Recurrence of parotid tumor; (3) Prior biopsy, chemotherapy, or radiotherapy before CT scanning. Image exclusion criteria were: (1) Significant artifacts on CT images; (2) Tumors predominantly cystic (over 70%); (3) Invasion of tumors into neighboring tissues; (4) Lymphatic metastasis. Ultimately, 218 patients from Hospitals 1, 2, and 3 were designated for the training set, and 32 patients from Hospitals 4 and 5 for the independent testing set. Within the training set, patients were randomly divided into a training and validation set in an 8:2 ratio. The patient recruitment process is detailed in **Figure 1A**. This research was approved by the Institutional Research Ethics Committee of the First Peoples' Hospital of Foshan [approval no. 2021 (2th)].

### CT image acquisition

The multiphase contrast-enhanced CT scans were conducted using advanced equipments: GE Gemstone Spectrum CT (Discovery 750HD, USA), 256Brilliance iCT (Philips, Netherlands), and Siemens SOMATOM Definition Flash (Siemens, Germany). The scanning parameters were standardized at 200mA, 120kVp, with a tube rotation time:0.6s, field of view (FOV):16cm×16cm, matrix resolution:512×512, and slice thickness: 1.25mm. Each patient underwent scanning at the lesion site, encompassing the entire parotid gland. Following the maxillofacial scan, double-phase enhancement scanning was initiated. For contrast, ioversol (350mg I/mL) or iopamidol injection(370mg I/mL), the nonionic contrast medium, was administered intravenously through the forearm’s elbow at a rate of 1 mL/kg body weight and an injection speed of 3.5 mL/s. The arterial and venous phases of enhancement were captured at intervals of 25–35 seconds and 50–60 seconds, respectively, post-contrast administration.

### Clinical characteristic and radiological features assessment

The clinical characteristics of the patients, including age and gender, were meticulously recorded. Two experienced radiologists (Radiologist 1 with 5 years of experience and Radiologist 2 with 10 years of experience in head and neck imaging diagnosis) who were blinded to the pathologic diagnosis review all the CT images to evaluate the radiological features. Approximately 7% (about 15 cases) of cases were controversial in terms of clinical and imaging characteristics, and consensus was reached through consultation between two radiologists. The findings of CT images as following were affirmed. (1) scope (unilateral or bilateral); (2) location (deep lobe, superficial lobe or both); (3) mean diameter of the tumor on axial CT imaging; (4) max diameter of the tumor on axial CT imaging; (5) calcification (absent or present); (6) margin (well-defined or ill-defined); (7) shape (regular or irregular); (8) cystic (absent or present); (9) vessel facing sign (absent or present).

### Image segmentation and radiomic features extraction

The volume of interest (VOI) for 3D analysis was meticulously delineated on axial CT images across the plain phase, arterial phase, and venous phase using the ITK-SNAP software (version 3.6.0) by Radiologist 1. To ensure the precision and reliability of feature extraction, Radiologist 2 conducted a thorough review of the VOI. Both radiologists conducted their assessments without knowledge of the pathological outcomes. In case of disagreements (20/250 lesions), a resolution was sought through dialogue and negotiation to definitively establish the VOI. Pre-processing, including voxel size normalization and image resampling, was also performed prior to feature extraction. The synthetic minority oversampling technique (SMOTE) algorithm was used to balance minority samples in the training set at a 1:1 ratio.

A comprehensive suite of 1,834 radiomics features was derived from the handcrafted VOI for each phase. These features were organized into three main categories, each reflecting a unique aspect of the tumor’s properties: (1) Geometry Features: This category encompasses metrics that detail the tumor’s three-dimensional shape and structural attributes, providing insight into its geometric dimensions. (2) Intensity Features: Focused on the first-order statistical analysis of voxel intensities within the tumor, this category offers a quantitative evaluation of the tumor’s radiographic intensity. (3) Texture Features: Essential for illustrating the voxel intensity patterns, including second-order and higher spatial distributions, this category employs several analytical techniques such as the Gray-Level Co-occurrence Matrix (GLCM), Gray-Level Run Length Matrix (GLRLM), Gray-Level Size Zone Matrix (GLSZM), and Neighborhood Gray-Tone Difference Matrix (NGTDM).This structured approach facilitates a detailed examination of the tumor’s characteristics, enhancing the understanding of its complex radiomic profile.

### Radiomics feature selection and radiomics signature construction

The Mann-Whitney U test was utilized to evaluate the significance of each radiomic feature, with a significance threshold established at a p-value of less than 0.05. Only features meeting this criterion were retained. For those exhibiting high repeatability, Spearman’s rank correlation coefficient was employed for correlation analysis, aiding in the detection of strongly correlated features. To minimize redundancy, a greedy recursive deletion strategy was implemented. During this procedure, from any pair of features with a correlation coefficient exceeding 0.9, one feature was preserved while the one deemed most redundant was eliminated in successive iterations.

The construction of the radiomic signature involved using the Least Absolute Shrinkage and Selection Operator (LASSO) regression model on the discovery dataset. LASSO, by modulating the regularization weight (λ), compresses all regression coefficients toward zero, effectively eliminating many irrelevant features by setting their coefficients to zero. To identify the optimal λ value, we employed 10-fold cross-validation with a minimum criteria method, choosing the λ that resulted in the lowest cross-validation error. The final radiomic signature was composed of features with non-zero coefficients, integrated into a linear model. The radiomic score for each patient was computed as a linear combination of these retained features, each weighted according to its model coefficient. We used the Python scikit-learn package for the LASSO regression modeling. Following the feature selection by LASSO, a Light Gradient Boosting Decision Tree (LightGBM) model was developed to differentiate between PAs and PCAs based on the training dataset. LightGBM is a histogram-based decision tree algorithm with differential acceleration based on the histogram. It uses a grow-by-leaf algorithm with depth limitation to improve the efficiency of training and prevent model overfitting (21). The diagnostic performance of this model, denoted as the radiomic signature, was outlined. The radiomics analysis workflow is illustrated in **Figure 1B**.

### Clinical model and radiomics nomogram construction

We conducted a comparative analysis of clinical factors, encompassing both clinical characteristics and radiological findings, between atypical PCAs and PAs. This comparison utilized both univariate and multivariate logistic regression analyses. Each independent factor’s predictive power for relative risk was assessed by calculating odds ratios (OR) with 95% confidence intervals (CIs). Significant clinical factors (p<0.05) identified through univariate logistic regression analysis were selected to construct a clinical model.

To enhance the intuitive and efficient assessment of the added prognostic value of a radiomics signature alongside clinical risk factors, we developed a comprehensive radiomics nomogram. This tool integrates significant clinical factors with the radiomics signature for a more holistic evaluation. We rigorously assessed the performance and reliability of each model — the clinical model, the radiomics signature, and the radiomics nomogram— by calculating their accuracy, precision, recall, F1 score and the area under the receiver operating characteristic (ROC) curve (AUC) across three distinct sets: the training set, the validation set, and an independent testing set. The DeLong test was utilized for a detailed comparison of their respective performances. Furthermore, to gauge the clinical utility of these predictive models, we conducted Decision Curve Analysis (DCA) and calibration curves. This approach measures the net benefits at various threshold probabilities, thus providing a comprehensive perspective on the clinical applicability and advantage of the models. Additionally, we implemented the Shapley additive explanation (SHAP) methodology to meticulously analyze the impact of individual variables on predictions within the independent testing set. The significance of each feature was scrutinized and ranked based on their mean SHAP values in descending order, offering insights into their relative contributions to the predictive accuracy.

### Statistical analysis

In this study, Python version 3.7.12 was utilized for feature extraction, feature selection, and model development. Quantitative variables were presented as means ± standard deviation (SD) or as medians with interquartile ranges (IQR). Meanwhile, categorical variables were reported as frequencies. A comparative analysis of the clinical characteristics of our patient cohort was conducted using a range of statistical tests. This included the independent sample t-test, the Mann-Whitney U test, and the Chi-square test, each selected based on its suitability for the type and distribution of the data. Differences in the AUC between various models were evaluated using the DeLong test. A p-value of less than 0.05 was considered statistically significant.

## Result

### Clinical-radiological characteristics and clinical model

The clinical characteristics and radiological findings of patients across the training, validation, and independent testing sets are meticulously outlined in **Table 1**. Notable differences were observed in the mean diameters of tumors, as well as their margins, between PAs and atypical PCAs across all datasets. **Table 2** presents a logistic regression analysis (LRA) focusing on various clinical risk factors, which includes both clinical characteristics and radiological findings. LightGBM-based clinical models were developed using clinically and significant radiological features identified in the univariate analysis. These features encompass age, tumor margin, location, shape, maximum diameter, mean diameter, the vessel facing sign, and calcification. The diagnostic efficacy of these clinical models is demonstrated in **Table 3**. Regarding the LightGBM-based clinical model: in the training set, it achieved an AUC of 0.869 (95% Confidence Interval [CI]= 0.805-0.933) with an accuracy of 81.0%; in the validation set, the AUC was 0.673 (95% CI= 0.456-0.890) with an accuracy of 75.0%; and in the independent testing set, the AUC was 0.746 (95% CI= 0.554-0.938) with an accuracy of 0.781%.

**Table 1:** Clinical characteristic and radiological findings of PAs and Atypical PCAs.

Feature	Training set			Validation set			Independent testing set		
	PA	PCA	p	PA	PCA	p	PA	PCA	p
Age	42.88 ± 14.37	47.66 ± 17.12	0.072	42.69 ± 13.73	50.08 ± 20.55	0.174	48.05 ± 14.80	49.17 ± 22.34	0.866
Dmean*	21.68 ± 6.90	24.74 ± 9.36	0.022	20.03 ± 7.09	25.62 ± 7.18	0.025	19.23 ± 5.40	30.33 ± 15.62	0.007
Dmax*	24.21 ± 8.40	27.18 ± 10.50	0.059	22.06 ± 8.00	29.58 ± 8.37	0.009	21.05 ± 6.34	28.62 ± 12.87	0.033
Gender			0.718			0.770			0.555
female	74 (56.92)	23 (52.27)		22 (68.75)	7 (58.33)		15 (75.00)	7 (58.33)	
male	56 (43.08)	21 (47.73)		10 (31.25)	5 (41.67)		5 (25.00)	5 (41.67)	
Scope			0.552			1.000			1.000
unilateral	126 (96.92)	44 (100.00)		32 (100.00)	12 (100.00)		20 (100.00)	12 (100.00)	
bilateral	4 (3.08)	0		0	0		0	0	
Location			0.028			0.072			0.018
Superficial lobe	86 (66.15)	20 (45.45)		20 (62.50)	5 (41.67)		12 (60.00)	3 (25.00)	
Deep lobe	10 (7.69)	3 (6.82)		1 (3.12)	3 (25.00)		5 (25.00)	9 (75.00)	
both	34 (26.15)	21 (47.73)		11 (34.38)	4 (33.33)		3 (15.00)	0	
Calcification			0.467			0.024			0.639
absent	124 (95.38)	40 (90.91)		32 (100.00)	9 (75.00)		19 (95.00)	10 (83.33)	
present	6 (4.62)	4 (9.09)		0	3 (25.00)		1 (5.00)	2 (16.67)	
Margin*			<0.001.000			0.015			0.007
ill-defined	18 (13.85)	27 (61.36)		7 (21.88)	8 (66.67)		4 (20.00)	9 (75.00)	
well-defined	112 (86.15)	17 (38.64)		25 (78.12)	4 (33.33)		16 (80.00)	3 (25.00)	
Shape			0.046			0.973			0.212
regular	50 (38.46)	9 (20.45)		10 (31.25)	3 (25.00)		9 (45.00)	2 (16.67)	
irregular	80 (61.54)	35 (79.55)		22 (68.75)	9 (75.00)		11 (55.00)	10 (83.33)	
Cystic			0.147			1.000			0.530
absent	36 (27.69)	18 (40.91)		11 (34.38)	4 (33.33)		2 (10.00)	3 (25.00)	
present	94 (72.31)	26 (59.09)		21 (65.62)	8 (66.67)		18 (90.00)	9 (75.00)	
Vessel facing sign			0.055			0.584			0.258
absent	115 (88.46)	33 (75.00)		28 (87.50)	9 (75.00)		20 (100.00)	10 (83.33)	
present	15 (11.54)	11 (25.00)		4 (12.50)	3 (25.00)		0	2 (16.67)	

Quantitative data are presented as mean ± standard deviation, p value was calculated with Student’s t-test or Mann-Whitney U test. Categorical variables are expressed as numbers (percentage), p value was calculated with the chi-square test or Fisher’extract test. PA, parotid polymorphic adenomas; PCA, parotid carcinomas. Dmean, the mean diameter of the tumor on axial CT imaging; Dmax, the max diameter of the tumor on axial CT imaging *p<0.05.

**Table 2:** Logistic regression analysis of clinical and CT features in training set.

Clinical-CT imaging features	Univariable analysis		Multivariable analysis	
	OR	p	OR	p
Age	1.004 (1.001,1.008)	0.024	1.004 (1.001,1.007)	0.023
Gender	1.046 (0.946,1.155)	0.460	NA	NA
Margin	0.637 (0.578,0.703)	<0.001	0.665 (0.600,0.736)	<0.001
Scope	0.770 (0.535,1.108)	0.237	NA	NA
Location	1.123 (1.041,1.212)	0.012	1.008 (0.933,1.089)	0.858
Shape	1.144 (1.031,1.269)	0.032	1.050 (0.952,1.157)	0.408
Cystic	0.913 (0.822,1.014)	0.156	NA	NA
Dmax	1.009 (1.004,1.015)	0.005	0.989 (0.968,1.011)	0.424
Dmean	1.012 (1.005,1.018)	0.002	1.016 (0.990,1.042)	0.323
Vessel facing sign	1.218 (1.064,1.394)	0.017	1.095 (0.965,1.244)	0.239
Calcification	1.349 (1.100,1.655)	0.016	1.098 (0.907,1.330)	0.422

NA, Not available.

**Table 3:** Diagnostic performance of the clinical model, radiomics signature and radiomic nomogram.

Model and metric	AUC (95%CI)	Accuracy	Recall	F1 Score
Traning set (n=175)				
Clinical Model	0.869 (0.805 - 0.933)	0.810	0.862	0.872
Radiomic Signature: A	0.981 (0.969 - 0.993)	0.915	0.900	0.914
Radiomic Signature: A+P	0.994 (0.989 - 1.000)	0.962	0.946	0.961
Radiomic Signature: A+V	0.998 (0.995 - 1.000)	0.977	0.962	0.977
Radiomic Signature: A+P+V	0.998 (0.995- 1.0000)	0.985	0.992	0.985
Radiomic Nomogram	1.000 (1.000,1.000)	1.000	1.000	1.000
Validation set (n=43)				
Clinical Model	0.673 (0.456 - 0.890)	0.750	0.781	0.820
Radiomic Signature: A	0.759 (0.594 - 0.924)	0.682	0.719	0.767
Radiomic Signature: A+P	0.667 (0.489 - 0.844)	0.659	0.719	0.754
Radiomic Signature: A+V	0.698 (0.503 - 0.893)	0.705	0.750	0.787
Radiomic Signature: A+P+V	0.680 (0.471 - 0.888)	0.705	0.781	0.794
Radiomic Nomogram	0.854 (0.732, 0.976)	0.864	1.000	0.914
Independent testing set (n=32)				
Clinical Model	0.746 (0.554 - 0.938)	0.781	0.800	0.821
Radiomic Signature: A	0.792 (0.634 - 0.950)	0.688	0.750	0.750
Radiomic Signature: A+P	0.708 (0.522 - 0.894)	0.719	0.850	0.791
Radiomic Signature: A+V	0.783 (0.597 - 0.969)	0.688	0.850	0.773
Radiomic Signature: A+P+V	0.588 (0.345 - 0.830)	0.688	0.850	0.773
Radiomic Nomogram	0.783 (0.583, 0.983)	0.750	0.950	0.826

AUC, area under the receiver operating characteristic curve; CI, confidence inerval; A, the arterial phase; P, the plain phase; V, the venous phase. Radiomic Nomogram: The nomogram was established based on the clinical risk factors and the radiomics features of the arterial phase and plain phase.

### Radiomics feature extraction, selection and radiomic models

In this research, an extensive array of 5679 handcrafted features was meticulously extracted from the plain, arterial, and venous phases. Following a rigorous screening and selection process, radiomic features from each phase were utilized to develop radiomic models employing the machine learning algorithm LightGBM. The diagnostic performance of these radiomic models is detailed in **Table 3**. The AUC values varied between 0.981 and 1.000 in the training set, 0.667 to 0.759 in the validation set, and 0.588 to 0.792 in the independent testing set. Notably, in the training set, the predictive accuracy of the radiomic models was significantly superior to that of the clinical models (p<0.05). However, in both the validation and independent testing sets, the radiomic models did not demonstrate a statistically significant enhancement in diagnostic performance.

The radiomic models, which were incorporated in various phases, are presented in **Table 3**. For the single-phase models, the AUC for those based on the arterial phase were 0.981(95%CI= 0.969-0.993),0.759 (95%CI= 0.594-0.924), and 0.792 (95% CI= 0.634-0.950) in the training, validation, and independent testing sets, respectively. In the case of multi-phase models, the radiomic models were constructed using a combination of arterial and other phases (either plain or venous). According to Delong’s test, in the training set, the combined arterial and plain phase model (model**_A+P_**), the model incorporating arterial and venous phases (model**_A+V_**), and the model incorporating arterial, plain, and venous phases (model**_A+P+V_**) demonstrated superior predictive performance compared to the model based solely on the arterial phase (model**_A_**), with p-values of 0.04, 0.03 and 0.02, respectively. However, there were no significant differences between the models in the validation set and the independent testing group(p>0.05). Therefore, upon further evaluation of recall and F1 scores, the model**_A+P_** showed better diagnostic performance in the independent testing set. We opted to integrate radiological features from the arterial and plain phases with clinical risk factors for establishing a radiomic nomogram.

### The radiomics nomogram development and performance evaluation

**Figure 2:**
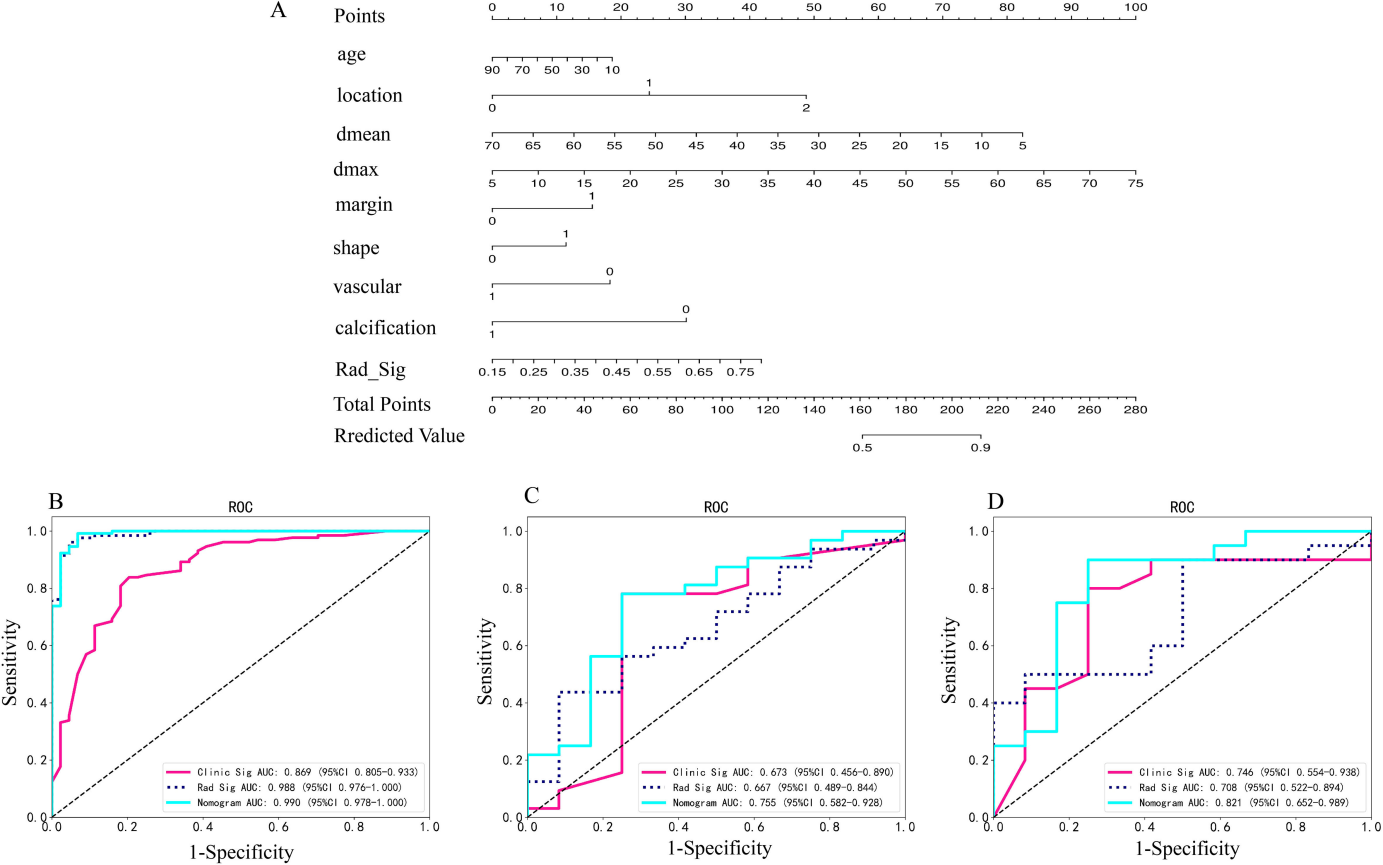
**(A)** Radiomics nomogram based on clinical risk factors and radiomics signature for distinguishing PAs and atypical PCAs. Comparison of ROC curve of clinical models, radiomic signatures and radiomic nomogram based on radiomics features in the arterial and plain phase in the training **(B)**, invalidation **(C)** and independent testing set **(D)**. *Rad-signature, radiomics signature; vascular, vessel facing sign; Dmean, the mean diameter of the tumor on axial CT imaging; Dmax, the max diameter of the tumor on axial CT imaging. *location, 0 means superficial lobe, 1 means superficial-deep lobe, 2 means deep lobe; margin, 0 means ill-defined, 1 means well-defined; shape, 0 means regular, 1 means irregular; vessel facing sign, 0 means absent, 1 means present; calcification, 0 means absent, 1 means present.

A radiomics nomogram, developed using the Logistic Regression algorithm, integrates clinical risk factors with 20 distinct radiomics features derived from arterial and plain phases, as depicted in **Figure 2A**. This nomogram reveals that the maximum and mean tumor diameters, location and margin of tumors, along with the radiomics score, significantly contribute to differentiating atypical PCAs from PAs, overshadowing other clinical risk factors in importance. The comparative diagnostic performances of various models are illustrated in **Figures 2B–D**. Notably, in the training set, the AUC values for the radiomics nomogram surpassed those of the clinical model (p=0.002). Similarly, in the independent testing set, the nomogram’s AUC was superior to that of the radiomics signature (p=0.024).

**Figure 3:**
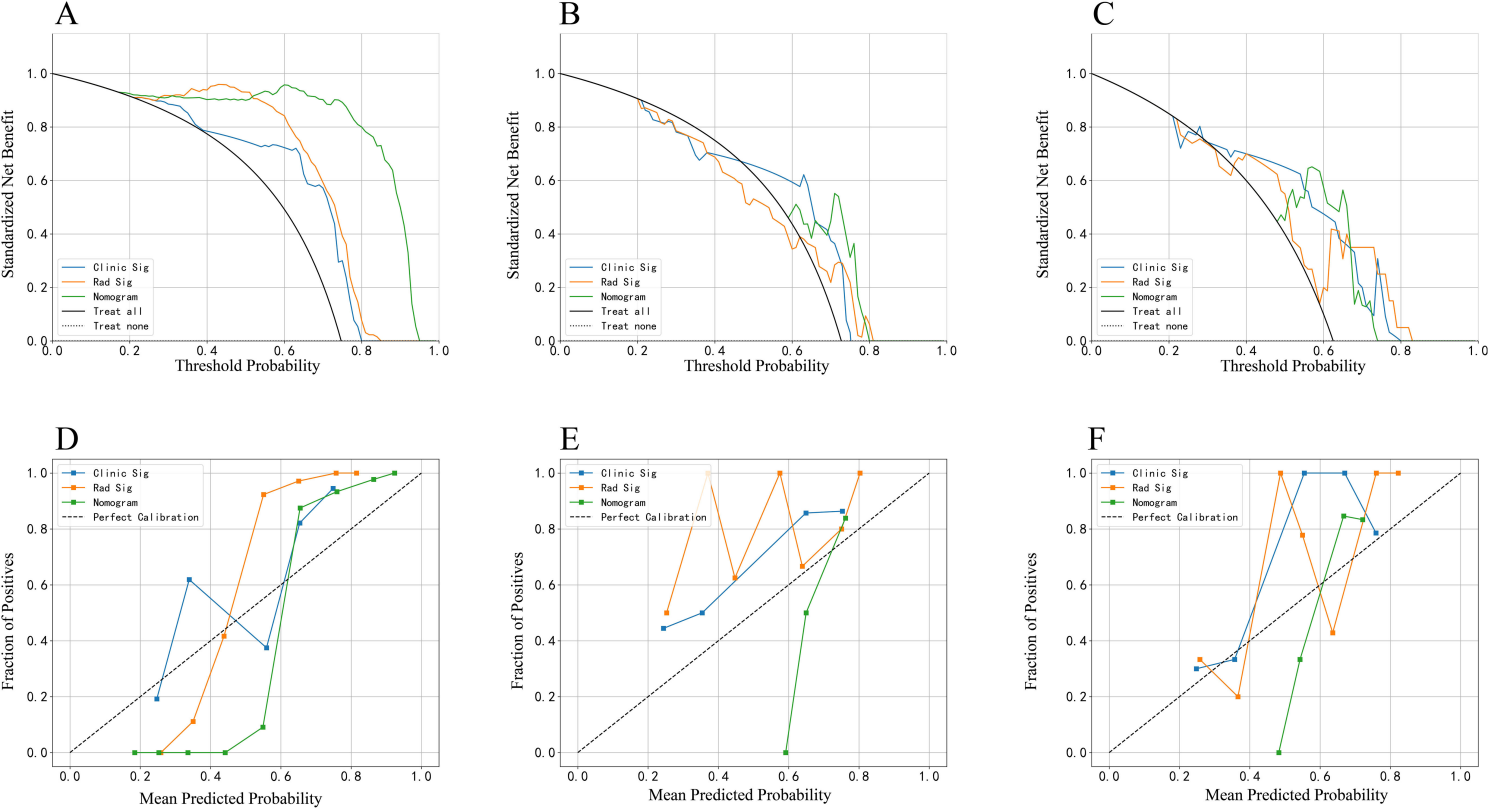
The decision curve analysis (DCA) curve and calibration curves of clinical models, radiomics signature and radiomics nomogram based on radiomics features in the arterial and plain phase. The DCA curve in the training **(A)**, invalidation **(B)** and independent testing **(C)** set. The calibration curves in the training **(D)**, invalidation **(E)** and independent testing **(F)** set.

**Figure 4:**
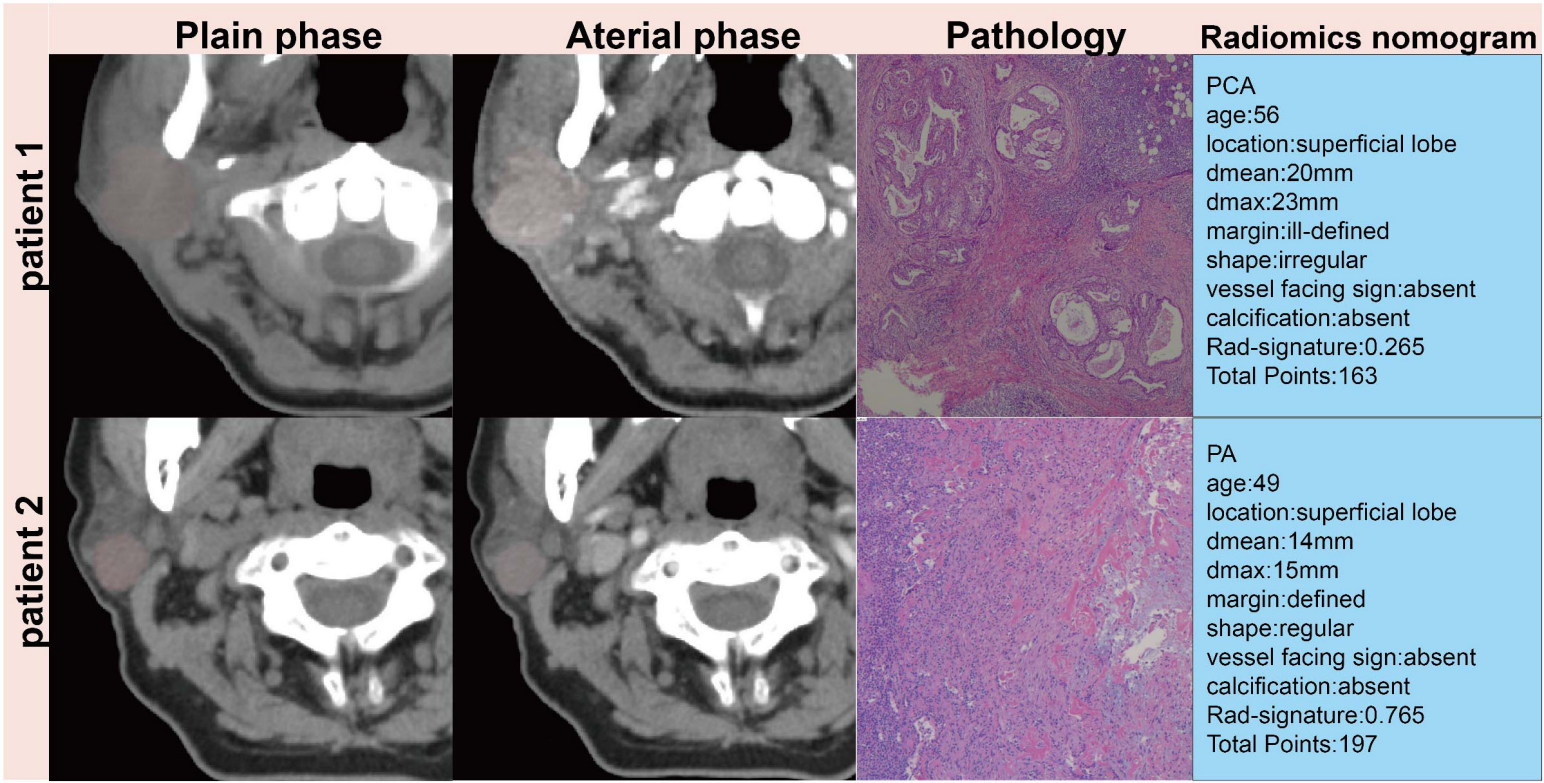
Patient 1, a 56 year-old female had a 23mm×17mm mass in the superficial lobe of the parotid gland which was PCA by pathology. Patient 2, a 49 year-old female had a 15×13mm mass in the superficial lobe of the parotid gland which was PA by pathology.

Decision Curve Analysis (DCA), as shown in **Figures 3A–C**, indicates that the radiomics nomogram offers greater clinical benefit compared to both the clinical model and the radiomics signature alone. Additionally, the calibration curves in **Figures 3D–F** demonstrate the nomogram’s effective performance in distinguishing between PAs and atypical PCAs. **Figure 4** showed the examples of radiomics nomogram models used in clinical practices for atypical PCA and PA discrimination. According to the nomogram, Patient 1, a 56 year-old female had a total score of 163, which was much lower than that of Patient 2 (total points 197). The risk of being diagnosed with PAs in Patient 1 and Patient 2 was 0.55 and 0.85, respectively. Finally, Patient 1 was pathologically confirmed to be PCA, while Patient 2 was pathologically confirmed to be PA.

### Interpretation of the radiomics nomogram model

**Figure 5:**
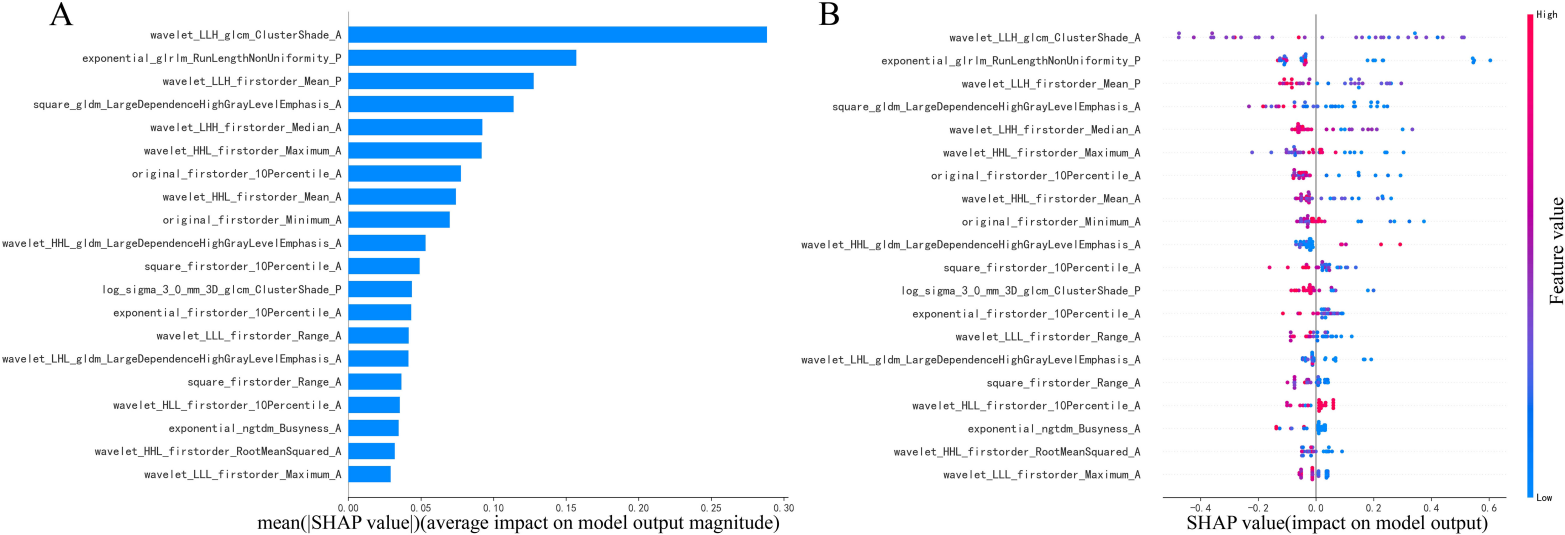
Illustration of radiomics features **(A, B)** in combination of arterial and plain phases with diagnosing PAs or atypical PCAs based on the LightGBM model using SHAP analysis.

The summary plot, shown in **Figure 5**, uses SHAP values to demonstrate the influence of various features on the model’s predictions for each patient. In this plot, individual points represent patients. The points change color from blue to red, with red indicating higher feature values and blue representing lower ones. The position of these points along the horizontal axis indicates the SHAP value, where a positive SHAP value suggests a higher likelihood of an atypical PCA diagnosis and a negative value leans towards a PA diagnosis. The placement of each point on the x-axis shows how much the corresponding feature impacts the prediction for an individual patient. Particularly, the cluster shade, run length non-uniformity, and first-order mean features extracted from images processed with wavelet or exponential filters, markedly differentiates atypical PCAs from PAs.

## Discussion

This study explores the development of more efficient diagnostic methods to differentiate between atypical PCAs and PAs. To achieve this, clinical models, a radiomics signature, and a radiomics nomogram were constructed. These tools were based on the clinical and radiomics features derived from multiphase CT images, utilizing the LightGBM model. The radiomics nomogram demonstrated exceptional diagnostic value, evidenced by its AUC scores of 1.000, 0.854, and 0.783, accuracy rates of 1.000, 0.864, and 0.750, and F1 score of 1.000,0.914 and 0.826 in the training, validation, and independent testing sets, respectively. Consequently, the radiomics nomogram shows great promise as an effective and non-invasive method for distinguishing atypical PCAs from PAs.

Although PAs and PCAs have well-defined classifications of benign and malignant tumors, but the diverse proportions of epithelial and mesenchymal components contribute to the varied morphological characteristics of PAs. In addition, the morphology of MPTs varies greatly among different tissue types. This complexity often makes it challenging to distinguish PCAs from PAs, particularly PAs with unclear margins and lobulated borders, atypical PCAs with clear boundaries, small size, no invasion of adjacent tissues or lymph node metastases, when using conventional CT (22). In this study, patients with invasion of adjacent tissues and lymph node metastasis were excluded, which increased the difficulty of differential diagnosis and had more clinical significance.

The results showed that age, tumor margins, shape, location and size were statistically different between PAs and atypical PCAs in training set. This explains the good diagnostic performance of the clinical model. Our study demonstrates that a clinical model, integrating clinical risk factors and radiological findings, performs satisfactorily, exhibiting AUC values of 0.869, 0.673, and 0.746 in training, validation, and independent testing sets, respectively. In previous studies, the patients with MPTs were elder than those with BPTs, and the sizes of MPTs were larger than BPTs (4). PAs occurred more often in women with the distribution of bimodal age of 38 and 64 (23). The significant radiological findings to indicating MPTs was lobular or irregular contour and ill-defined margin of the mass (24, 25). However, the above image feature extraction is subjective and greatly affected by the knowledge and experience of radiologists. A subspecialist with 30 years of experience in head and neck radiology demonstrated higher sensitivity and comparable specificity to MR radiomics models for PAs diagnosis (26). However, radiomics models potentially reduce the technical barriers in diagnosing parotid tumors.

The radiomics data and features derived from artificial intelligence algorithms quantify tumor heterogeneity, which may not be discernible to the naked eye, thereby enhancing the diagnostic utility of image datasets (27). CT/MR Radiomics has been widely used in the diagnosis of head and neck tumors, gene expression, immune status and efficacy evaluation, etc. In recent years, it has also been partially reported in the diagnosis of PGTs.MR-based radiomics achieves an accuracy of 80.4% in differentiating MPTs from BPTs (28) and 80.43% in distinguishing MPTs from PAs (29). He’s research indicated that the accuracy of the XGBoost model, based on MR images for differentiating MPTs from BPTs, surpasses that of radiologists (89.9% vs 83.1%) (30). Although MRI offers detailed insights, CT scans remain the preferred preoperative examination due to their lower cost, faster scanning, and fewer contraindications. Additionally, MRI- and CT-based radiomics signatures exhibit no significant disparity in differentiating PAs from WTs (31). Qiang discovered that the radiomics model, which combines three-phase enhanced CT imaging, particularly the arterial phase, significantly aids in diagnosing BPTs from MPTs (AUC=0.933, 0.936) (32). The support vector machine (SVM)-based prediction model reported AUCs of 0.854 in the training set and 0.741 in the test set in Xu’s study (33). These studies were constrained by the absence of an independent testing set and the small sample size of MPTs. Our study, incorporating patients from five different institutions, enhances the reliability and reproducibility of our results through verification in both validation and independent testing sets.

Previous researches utilized multiphasic CT scanning to analyze the enhancement patterns of PGTs, which are indicative of their pathological characteristics. There exists a debate over the optimal phase of enhancement to effectively differentiate between BPTs and MPTs. A notable observation is the limited efficacy of delayed enhancement in distinguishing PAs from PCAs (10, 34). However, findings from early contrast phase CT images (35) and radiomics models (19) have demonstrated superior performance in differentiating PCAs from PAs. These may be due to pathological features of PCAs: increased number of blood vessels, increased vascular permeability and abnormal arteriovenous fistula (36). Consequently, our study developed a radiomics model (model**_A_**) using the arterial phase features as the basic model. Additionally, we evaluated radiomics models that incorporated combinations of different phases. In the training set, radiomics signatures model**_A+P,_** model**_A+V_** and model**_A+P+v_** exhibited enhanced predictive capabilities compared to model**_A_**. The model**_A+P_** demonstrated superior diagnostic performance in the external test group, as indicated by its higher recall and F1 score, suggesting that the venous phase contributes marginally to the diagnosis of PGTs in radiomics model. This finding aligns with previous studies, where radiomics models based on the arterial phase outperformed those using the venous and plain phases in a single-phase analysis. Moreover, the best predictive performance was achieved by models that integrated all phases(model**_A+P+v_)** (32 **)**. Our radiomics analysis indicates the potential to optimize multiphase CT protocols for parotid tumor assessment. The high discriminative power of the model**_A+P_** for differentiation of PA and atypical PCA suggests that reduced protocols may maintain diagnostic accuracy while addressing critical limitations. This approach promises significant radiation reduction, which is particularly vital for head and neck imaging under the As Low As Reasonably Achievable (ALARA) principles, as well as enhanced clinical feasibility through shorter scan times. Importantly, this approach enables the retrospective application of radiomics to the abundant clinical CT archives containing incidentally detected parotid masses from routine examinations, substantially expanding its real-world applicability. However, future validation is necessary to confirm performance parity across multicenter cohorts with varying acquisition parameters. Both the arterial phase and the multiphase combined radiomics models demonstrated excellent and comparable effectiveness in differentiating PAs from other BPTs (19). Similarly, in a multicenter radiomics study, the arterial phase was selected for distinguishing PAs from WTs (18). In contrast to previous studies, our research innovatively combined radiomics signatures from plain or venous phases with those from the arterial phase, comparing these combinations to identify the most effective diagnostic approach for PGTs. The model_A+P_ demonstrated superior diagnostic performance, achieving a significantly higher AUC value in the training set compared to model**_A_**. While the AUC values for model_A+P_ remained higher than those for model**_A_** in both the validation and independent testing sets, the differences were not statistically significant. This suggests that while model_A+P_ offers an improvement in diagnostic performance, further investigation is needed to fully understand the clinical impact of these findings.

To enhance the efficiency of the diagnostic model, we estimated radiomics nomograms based on clinical risk factors combined with radiomics signatures model**_A+P_**. This integrated approach yielded AUC values ranging from 0.750 to 1.000, significantly improving the differentiation between PAs and atypical PCAs compared to the standalone radiomics model in the independent set (p < 0.05). Previous studies corroborate our findings, showing that radiomics nomograms that include both clinical factors and a radiomic signature outperform models based solely on radiomic scores (37) or clinical factors (38). Our study aligns with these results, demonstrating the enhanced diagnostic performance of this comprehensive approach.

To underscore the significance of various features in the model’s predictions and to augment model interpretability, key elements of this model were identified using the SHAP interpretation methodology (39). This study highlighted the pivotal role of the cluster shade, run length non-uniformity, and first-order mean features through wavelet or exponential transformation in diagnosing PAs and atypical PCAs. These features are used to quantify image inhomogeneity and texture directionality, which may reflect tumor heterogeneity to some extent. Echoing findings from prior research (32), it was found that sphericity and wavelet features derived from multiphasic CT images are closely associated with the diagnosis of benign and malignant PGTs. Sphericity features quantitatively assess the tumor region’s roundness in comparison to a sphere (40), while wavelet features are believed to capture the tumor’s heterogeneity (41) and are valuable in indicating tumor proliferation (42). In other texture analysis studies, the Gray Level Co-occurrence Matrix (GLCM) features, which represent the spatial relationship between neighboring pixels or voxels, have also been shown to depict tumor heterogeneity. These features have been utilized in constructing radiomics models for differentiating MPTs from PAs (33) and other subtypes of BPTs (19, 43). However, due to differences in incidence rates, even though this study included data from five centers, there was still an imbalance in the proportion of PCA and PA. Consequently, the calculation of SHAP values may have underestimated the significance of PCA characteristics, resulting in a bias in the local interpretation of PCA. Expanding the sample size of parotid gland cancer cases in future studies may resolve this issue.

Consequently, based on existing literature, there are no universally acknowledged image features for model construction (20), and the repeatability and reliability of these models warrant further investigation in future research.

Despite the promising findings, our study has limitation which require attention. Ever though we collected the patients from 5 different institutions, the sample size for parotid carcinoma is still not enough because of the low incidence rate. Therefore, the subtypes of parotid carcinomas were unable to be taken into consideration. The small sample size of PCAs also has an impact on the generalizability of the constructed model. Therefore, the sample size, especially for PCAs, will be further increased in the next study. Second, due to the large number of sites included, differences in machine and scanning protocols may cause image instability. Finally, deep learning which may provide more efficient compared with the traditional machine learning should be applied in the following study.

## Conclusion

In conclusion, the radiomics nomogram developed in this study, leveraging data from both arterial and plain CT phases, exhibits superior performance in accurately distinguishing between atypical PCAs and PAs. Consequently, it holds significant promise as an indispensable tool for enhancing clinical decision-making, offering a pathway toward more tailored and effective management strategies for patients with parotid gland disorders. Looking ahead, the integration of this nomogram into clinical practice could revolutionize the diagnostic approach, encouraging further research and development in this promising field.

## Data Availability

The raw data supporting the conclusions of this article will be made available by the authors, without undue reservation.

## References

[1] Saravakos P Kourtidis S Hartwein J Preyer S. Parotid gland tumors: A multicenter analysis of 1020 cases. Increasing incidence of warthin’s tumor. Indian J Otolaryngol Head Neck Surg. (2022) 74:2033–40. doi: 10.1007/s12070-020-01981-z, PMID: 36452806 PMC9702007

[2] Lanišnik B Levart P Čizmarevič B Švagan M. Surgeon-performed ultrasound with fine-needle aspiration biopsy for the diagnosis of parotid gland tumors. Head Neck. (2021) 43:1739–46. doi: 10.1002/hed.26630, PMID: 33547678

[3] Seyhun N Doğan U Çalış ZAB Kaya MF Hasçiçek S Turgut S. The role of fine needle aspiration biopsy in deep lobe parotid tumors: Comparison of preoperative cytology and postoperative histopathologic results. Am J Otolaryngol. (2021) 42:102590. doi: 10.1016/j.amjoto.2020.102590, PMID: 33045535

[4] Lee DH Jung EK Lee JK Lim SC. Comparative analysis of benign and Malignant parotid gland tumors: A retrospective study of 992 patients. Am J Otolaryngol. (2023) 44:103690. doi: 10.1016/j.amjoto.2022.103690, PMID: 36473266

[5] Galli A Tulli M Giordano L Biafora M Di Santo D Bondi S. Fine needle aspiration cytology for parotid neoplasms: risk of Malignancy through inconclusive results and lower grade tumors. Eur Arch Otorhinolaryngol. (2020) 277:841–51. doi: 10.1007/s00405-019-05733-w, PMID: 31745630

[6] Schmidt RL Hall BJ Wilson AR Layfield LJ. A systematic review and meta-analysis of the diagnostic accuracy of fine-needle aspiration cytology for parotid gland lesions. Am J Clin Pathol. (2011) 136:45–59. doi: 10.1309/ajcpoie0cznat6sq, PMID: 21685031

[7] Psychogios G Vlastos I Tholken R Zenk J. Warthin’s tumour seems to be the most common benign neoplasm of the parotid gland in Germany. Eur Arch Otorhinolaryngol. (2020) 277:2081–4. doi: 10.1007/s00405-020-05894-z, PMID: 32189070

[8] Eveson JW Cawson RA. Salivary gland tumours. A review of 2410 cases with particular reference to histological types, site, age and sex distribution. J Pathol. (1985) 146:51–8. doi: 10.1002/path.1711460106, PMID: 4009321

[9] Xu ZF Yong F Yu T Chen YY Gao Q Zhou T. Different histological subtypes of parotid gland tumors: CT findings and diagnostic strategy. World J Radiol. (2013) 5:313–20. doi: 10.4329/wjr.v5.i8.313, PMID: 24003357 PMC3758499

[10] Reginelli A Clemente A Renzulli M Maggialetti N Santagata M Colella G. Delayed enhancement in differential diagnosis of salivary gland neoplasm. Gland Surg. (2019) 8:S130–s135. doi: 10.21037/gs.2019.03.03, PMID: 31559179 PMC6755945

[11] Kong X Li H Han Z. The diagnostic role of ultrasonography, computed tomography, magnetic resonance imaging, positron emission tomography/computed tomography, and real-time elastography in the differentiation of benign and Malignant salivary gland tumors: a meta-analysis. Oral Surg Oral Med Oral Pathol Oral Radiol. (2019) 128:431–443.e1. doi: 10.1016/j.oooo.2019.06.014, PMID: 31327623

[12] Huang N Chen Y She D Xing Z Chen T Cao D. Diffusion kurtosis imaging and dynamic contrast-enhanced MRI for the differentiation of parotid gland tumors. Eur Radiol. (2022) 32:2748–59. doi: 10.1007/s00330-021-08312-y, PMID: 34642805 PMC8921043

[13] Takumi K Nagano H Kikuno H Kumagae Y Fukukura Y Yoshiura T. Differentiating Malignant from benign salivary gland lesions: a multiparametric non-contrast MR imaging approach. Sci Rep. (2021) 11:2780. doi: 10.1038/s41598-021-82455-2, PMID: 33531644 PMC7854671

[14] Le NQK Kha QH Nguyen VH Chen YC Cheng SJ Chen CY. Machine learning-based radiomics signatures for EGFR and KRAS mutations prediction in non-small-cell lung cancer. Int J Mol Sci. (2021) 22(17):9254. doi: 10.3390/ijms22179254, PMID: 34502160 PMC8431041

[15] Conti A Duggento A Indovina I Guerrisi M Toschi N. Radiomics in breast cancer classification and prediction. Semin Cancer Biol. (2021) 72:238–50. doi: 10.1016/j.semcancer.2020.04.002, PMID: 32371013

[16] Zhong ME Duan X Ni-Jia-Ti MY Qi H Xu D Cai D. CT-based radiogenomic analysis dissects intratumor heterogeneity and predicts prognosis of colorectal cancer: a multi-institutional retrospective study. J Transl Med. (2022) 20:574. doi: 10.1186/s12967-022-03788-8, PMID: 36482390 PMC9730572

[17] Zheng YM Xu WJ Hao DP Liu XJ Gao CP Tang GZ. A CT-based radiomics nomogram for differentiation of lympho-associated benign and Malignant lesions of the parotid gland. Eur Radiol. (2021) 31:2886–95. doi: 10.1007/s00330-020-07421-4, PMID: 33123791

[18] Feng B Wang Z Cui J Li J Xu H Yu D. Distinguishing parotid polymorphic adenoma and warthin tumor based on the CT radiomics nomogram: A multicenter study. Acad Radiol. (2023) 30:717–26. doi: 10.1016/j.acra.2022.06.017, PMID: 35953356

[19] Chen F Ge Y Li S Liu M Wu J Liu Y. Enhanced CT-based texture analysis and radiomics score for differentiation of pleomorphic adenoma, basal cell adenoma, and Warthin tumor of the parotid gland. Dentomaxillofac Radiol. (2023) 52:20220009. doi: 10.1259/dmfr.20220009, PMID: 36367128 PMC9974237

[20] Mao K Wong LM Zhang R So TY Shan Z Hung KF. Radiomics analysis in characterization of salivary gland tumors on MRI: A systematic review. Cancers (Basel). (2023) 15(20):4918. doi: 10.3390/cancers15204918, PMID: 37894285 PMC10605883

[21] Guolin Ke QM Finley T Wang T Chen W Ma W Ye Q. Lightgbm: A highly efficient gradient boosting decision tree. Adv Neural Inf. (2017) 30.

[22] Kim H Kim SY Kim YJ Ko JM Park MJ Kim JH. Correlation between computed tomography imaging and histopathology in pleomorphic adenoma of parotid gland. Auris Nasus Larynx. (2018) 45:783–90. doi: 10.1016/j.anl.2017.09.013, PMID: 29055657

[23] Valstar MH de Ridder M van den Broek EC Stuiver MM van Dijk BAC van Velthuysen MLF. Salivary gland pleomorphic adenoma in the Netherlands: A nationwide observational study of primary tumor incidence, Malignant transformation, recurrence, and risk factors for recurrence. Oral Oncol. (2017) 66:93–9. doi: 10.1016/j.oraloncology.2017.01.004, PMID: 28249655

[24] Kim KH Sung MW Yun JB Han MH Baek CH Chu KC. The significance of CT scan or MRI in the evaluation of salivary gland tumors. Auris Nasus Larynx. (1998) 25:397–402. doi: 10.1016/s0385-8146(98)00012-1, PMID: 9853663

[25] Christe A Waldherr C Hallett R Zbaeren P Thoeny H. MR imaging of parotid tumors: typical lesion characteristics in MR imaging improve discrimination between benign and Malignant disease. AJNR Am J Neuroradiol. (2011) 32:1202–7. doi: 10.3174/ajnr.A2520, PMID: 21724574 PMC7966029

[26] Vernuccio F Arnone F Cannella R Verro B Comelli A Agnello F. Diagnostic performance of qualitative and radiomics approach to parotid gland tumors: which is the added benefit of texture analysis? Br J Radiol. (2021) 94:20210340. doi: 10.1259/bjr.20210340, PMID: 34591597 PMC8631014

[27] Lambin P Rios-Velazquez E Leijenaar R Carvalho S van Stiphout RG Granton P. Radiomics: extracting more information from medical images using advanced feature analysis. Eur J Cancer. (2012) 48:441–6. doi: 10.1016/j.ejca.2011.11.036, PMID: 22257792 PMC4533986

[28] Zheng YM Li J Liu S Cui JF Zhan JF Pang J. MRI-Based radiomics nomogram for differentiation of benign and Malignant lesions of the parotid gland. Eur Radiol. (2021) 31:4042–52. doi: 10.1007/s00330-020-07483-4, PMID: 33211145

[29] Gabelloni M Faggioni L Attanasio S Vani V Goddi A Colantonio S. Can magnetic resonance radiomics analysis discriminate parotid gland tumors? A pilot study. Diagn (Basel). (2020) 10(11):900. doi: 10.3390/diagnostics10110900, PMID: 33153140 PMC7692594

[30] He Z Mao Y Lu S Tan L Xiao J Tan P. Machine learning-based radiomics for histological classification of parotid tumors using morphological MRI: a comparative study. Eur Radiol. (2022) 32:8099–110. doi: 10.1007/s00330-022-08943-9, PMID: 35748897

[31] Liu Y Zheng J Lu X Wang Y Meng F Zhao J. Radiomics-based comparison of MRI and CT for differentiating pleomorphic adenomas and Warthin tumors of the parotid gland: a retrospective study. Oral Surg Oral Med Oral Pathol Oral Radiol. (2021) 131:591–9. doi: 10.1016/j.oooo.2021.01.014, PMID: 33602604

[32] Yu Q Wang A Gu J Li Q Ning Y Peng J. Multiphasic CT-based radiomics analysis for the differentiation of benign and Malignant parotid tumors. Front Oncol. (2022) 12:913898. doi: 10.3389/fonc.2022.913898, PMID: 35847942 PMC9280642

[33] Xu Y Shu Z Song G Liu Y Pang P Wen X. The role of preoperative computed tomography radiomics in distinguishing benign and Malignant tumors of the parotid gland. Front Oncol. (2021) 11:634452. doi: 10.3389/fonc.2021.634452, PMID: 33777789 PMC7988088

[34] Jin GQ Su DK Xie D Zhao W Liu LD Zhu XN. Distinguishing benign from Malignant parotid gland tumours: low-dose multi-phasic CT protocol with 5-minute delay. Eur Radiol. (2011) 21:1692–8. doi: 10.1007/s00330-011-2101-y, PMID: 21547526 PMC3128264

[35] Zuo H. The clinical characteristics and CT findings of parotid and submandibular gland tumours. J Oncol. (2021) 2021:8874100. doi: 10.1155/2021/8874100, PMID: 34306079 PMC8272666

[36] Dvorak HF. Tumor stroma, tumor blood vessels, and antiangiogenesis therapy. Cancer J. (2015) 21:237–43. doi: 10.1097/ppo.0000000000000124, PMID: 26222073

[37] Muntean DD Dudea SM Baciut M Dinu C Stoia S Solomon C. The role of an MRI-based radiomic signature in predicting Malignancy of parotid gland tumors. Cancers (Basel). (2023) 15(13):3319. doi: 10.3390/cancers15133319, PMID: 37444429 PMC10340186

[38] Piludu F Marzi S Ravanelli M Pellini R Covello R Terrenato I. MRI-based radiomics to differentiate between benign and Malignant parotid tumors with external validation. Front Oncol. (2021) 11:656918. doi: 10.3389/fonc.2021.656918, PMID: 33987092 PMC8111169

[39] Marcílio WE Eler DM. From explanations to feature selection: assessing SHAP values as feature selection mechanism. 2020 33rd SIBGRAPI Conference on Graphics, Patterns and Images (SIBGRAPI). (2020) 340–7. Institute of Electrical and Electronic Engineers, Porto de Galinhas, Brazil. doi: 10.1109/SIBGRAPI51738.2020.00053

[40] van Griethuysen JJM Fedorov A Parmar C Hosny A Aucoin N Narayan V. Computational radiomics system to decode the radiographic phenotype. Cancer Res. (2017) 77:e104–7. doi: 10.1158/0008-5472.Can-17-0339, PMID: 29092951 PMC5672828

[41] Ji GW Zhu FP Xu Q Wang K Wu MY Tang WW. Radiomic features at contrast-enhanced CT predict recurrence in early stage hepatocellular carcinoma: A multi-institutional study. Radiology. (2020) 294:568–79. doi: 10.1148/radiol.2020191470, PMID: 31934830

[42] Liang W Yang P Huang R Xu L Wang J Liu W. A combined nomogram model to preoperatively predict histologic grade in pancreatic neuroendocrine tumors. Clin Cancer Res. (2019) 25:584–94. doi: 10.1158/1078-0432.Ccr-18-1305, PMID: 30397175

[43] Zhang D Li X Lv L Yu J Yang C Xiong H. Improving the diagnosis of common parotid tumors via the combination of CT image biomarkers and clinical parameters. BMC Med Imaging. (2020) 20:38. doi: 10.1186/s12880-020-00442-x, PMID: 32293304 PMC7161241

